# A Review of Advancement on Influencing Factors of Acne: An Emphasis on Environment Characteristics

**DOI:** 10.3389/fpubh.2020.00450

**Published:** 2020-09-17

**Authors:** Jianting Yang, Haoran Yang, Aie Xu, Li He

**Affiliations:** ^1^The Center for Modern Chinese City Studies, East China Normal University, Shanghai, China; ^2^School of Urban and Regional Science, East China Normal University, Shanghai, China; ^3^Department of Dermatology and Venereology, First Affiliated Hospital of Kunming Medical University, Kunming, China; ^4^Department of Dermatology, Third People's Hospital of Hangzhou, Hangzhou, China

**Keywords:** acne, sociology of population, natural environment, social environment, built environment, green spaces

## Abstract

**Background:** Acne vulgaris is known as a commonly-seen skin disease with a considerable impact on the quality of life. At present, there have been a growing number of epidemiological, medical, demographic and sociological researches focusing on various influencing factors in the occurrence of acne. Nevertheless, the correlation between environmental factors and acne has yet to be fully investigated.

**Objective:** To assess the impacts of individual, natural and social environmental factors on acne and to construct a framework for the potential impact of built environment on acne.

**Methods:** A thorough review was conducted into the published social demographical, epidemiological, and environmental studies on acne through PubMed, Google Scholar and Web of Science, with reference made to the relevant literature.

**Results:** The influencing factors in acne are classed into four major categories. The first one includes individual socio-economic and biological factors, for example, gender, age, economic level, heredity, obesity, skin type, menstrual cycle (for females), diet, smoking, cosmetics products, electronic products, sleep quality and psychological factors. The second one includes such natural environmental factors as temperature, humidity, sun exposure, air pollution and chloracne. The third one relates to social environment, including social network and social media. The last one includes built environmental factors, for example, population density, food stores, green spaces, as well as other built environment characteristics for transport. Acne can be affected negatively by family history, overweight, obesity, oily or mixed skin, irregular menstrual cycles, sugary food, greasy food, dairy products, smoking, the improper use of cosmetics, the long-term use of electronics, the poor quality of sleep, stress, high temperature, sun exposure, air pollution, mineral oils and halogenated hydrocarbons. Apart from that, there are also potential links between built environment and acne.

**Conclusions:** It is necessary to determine the correlation between the built environment and acne based on the understanding of the impact of traditional factors (sociology of population and environment) on acne gained by multidisciplinary research teams. Moreover, more empirical studies are required to reveal the specific relationship between built environment and acne.

## Introduction

Chinese cities face many health challenges posed by rapidly changing urban environments (e.g., air pollution, water pollution, zoning and mix use of land, reduction of vegetation coverage and growing population density) and lifestyles (e.g., lacking physical activity, unbalanced diets, tobacco and alcohol use), especially non-communicable chronic diseases, such as cardiovascular disease, cancer, respiratory diseases, diabetes and mental illness, which have replaced infectious diseases as major contributors to the overall disease burden (1). Many skin diseases are also non-communicable chronic diseases, especially acne that mainly occurs on the face, which is easily affected by external factors.

Acne is a chronic inflammatory skin disease involving the sebaceous glands. Four major pathogenesis are involved in the development of androgen-induced increased sebum hyperproduction, altered follicular keratinization, inflammation and Propionibacterium acnes (P. acne) (2, 3). It is also affected by environmental pollution, social environment, changes of dietary structure and lifestyle, for example, worsening air pollution, the intake of sweets, staying up late, social network and social media. Thus, the prevalence of acne increases year by year. According to a systematic analysis for the Global Burden of Disease Study, in 2010, the prevalence of acne among all the population in the world was 9.38%, ranking the eighth in the world (4). From 2006 to 2016, the prevalence of acne increased by 5.1% (5). In the meantime, in the US, the median cost per person per 7 months for acne treatments approved by the US Food and Drug Administration was $350–3,806^1^ (6). Because of its high prevalence and recurrence, acne patients have suffered from the corresponding economic burden. In addition, although acne is not a life-threatening disease, it damages the appearance, which might leave scars on patients if not treated in time. Moreover, for young men and women, discosmetic dermatosis can easily lead to inferiority, even affecting the employment and marriage of patients. According to the study in China, 30.8% acne patients reported that acne had a negative impact on their quality of life (7). Several studies showed that people with acne had lower self-confidence, the difficulty of making friends, challenges of going to school, and the trouble of finding a job (8, 9). Moreover, acne patients have a higher propensity of underlying mental disorders, including anxiety, depression and suicide (10, 11). The prevalence of acne can not only impact the cost of drug treatment, but also the psychological disorders associated with acne and quality of life.

Built environment is defined to include all buildings, spaces, and products that are built or modified by humans. There is growing evidence that the built environment affects health in different ways and mechanisms (12), especially chronic diseases such as obesity (13), mental health (14), cardiovascular disease (15), and respiratory health (16). Acne is a chronic disease in which both environmental and genetic factors interact (17). Therefore, it might also be affected by the built environment. However, previous acne epidemiological studies mainly focus on individual factors (such as family history, diet, lifestyle, occupation, and psychological factors) and other natural and social environmental factors (such as air pollution and social network) (18, 19), there is very limited research that examines whether a relationship exists between the built environment and acne. In order to fill up this gap, this article will first do a comprehensive review on the basis of the previous studies of sociology of population, epidemiology, and environmental factors, and further build a framework for the potential impact of built environment on acne for the future research.

## Search Strategies and Selection of Studies

**Figure 1:**
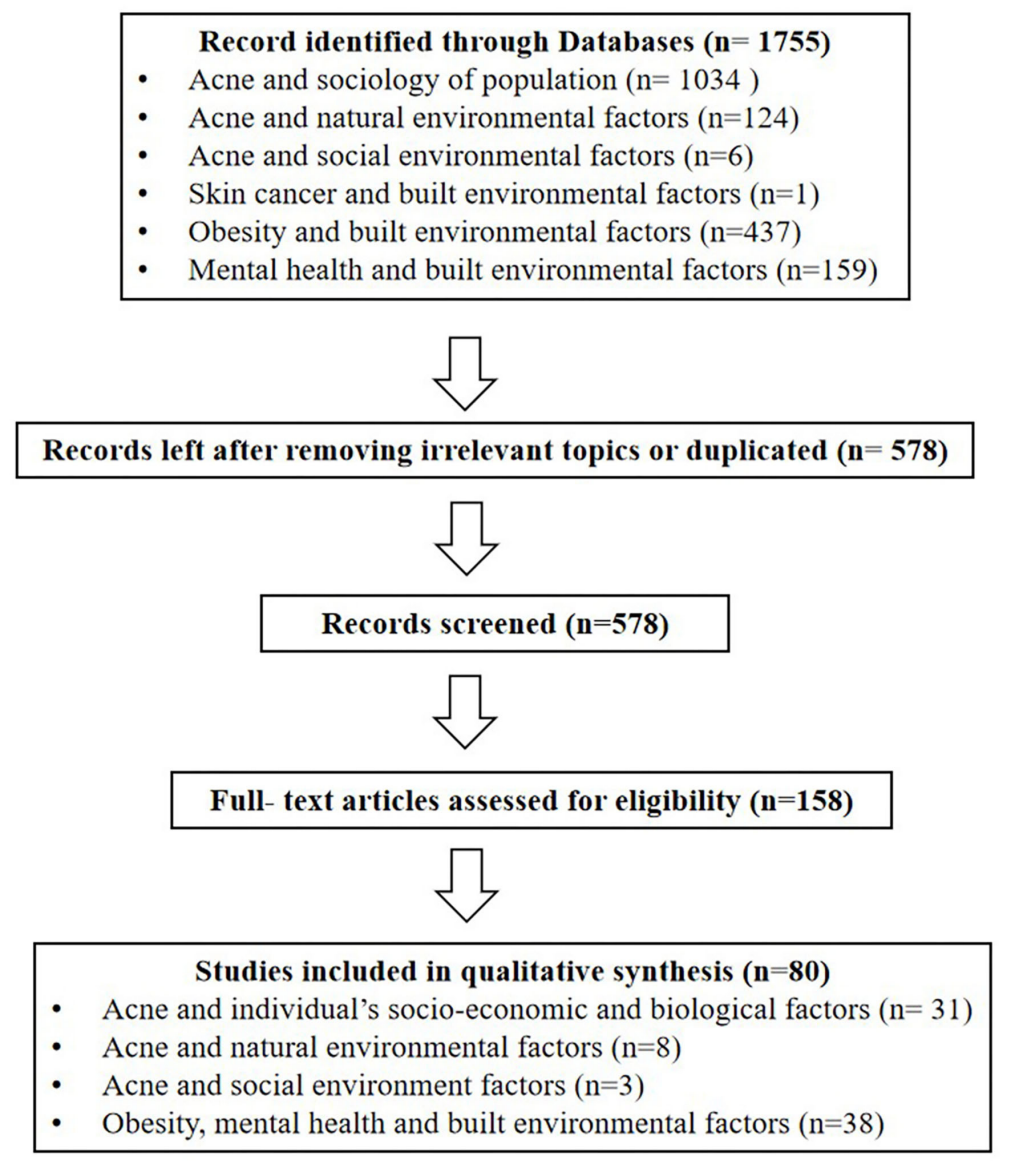
Illustrates the study inclusion and exclusion process.

We searched all publications included in the electronic databases of PubMed, Google Scholar and Web of Science (from 2000–present). The search stratagem used the term “acne vulgaris (or acne),” with the following combinations: epidemiology, prevalence, Propionibacterium acnes (or P. Acnes), sociology of population, gender, age, hormones, diet, sweets, milk, dairy, greasy, dairy products, spicy, chocolate, glycaemic index, smoking, tobacco, cosmetics, electronic productors, overweight, obesity, mental health, mental disorder, stress, economic, skin type, menstrual cycle, exposure, climate, environment, temperature, humidity, sun, pollution, chloracne, social environment, social network, social media, and built environment. Furthermore, in order to avoid missing relevant literature, we also reviewed the reference lists of the identified papers and manually searched for additional publications. Next, we evaluated the title and abstract of each article based on the inclusion criteria. The full text review was then conducted to determine whether the article met all criteria. Inclusion criteria included: (1) Being written in English, (2) Epidemiological studies of acne. Exclusion criteria included: (1) respondents with systemic disorders (such as cardiovascular, respiratory, urinary, reproductive and endocrine diseases, etc.)^2^. (2) studies that did not focus on acne. After searching the literature, there were few relevant studies on built environment and acne, only one study about built environment and skin cancer was found. Research found that the occurrence of acne was strongly attributed to the exposure of skin in the natural environment, the obesity and psychological issues, which could be affected by the built environment factors. In order to establish an indirect relationship between built environment and acne, the search stratagem also used the term “built environment,” with the following combinations: obesity, overweight, mental health, anxiety, depression and suicide. Inclusion criteria included: (1) Research written in English, (2) original articles. Exclusion criteria included: studies that did not have a significant focus on built environment and obesity, mental health. We initially selected 158 studies based on the titles and abstracts. After reading the full texts, a total of 80 articles met all the criteria and were included in the review. All the 80 studies identified were quantitative (Figure 1). Sample size ranged from 50 to 2472004.

## Results

The factors that affect acne are classified into four main categories: individual's socio-economic and biological factors, natural environmental factors, social environmental factors and built environmental factors.

### Individual's Socio-Economic and Biological Factors

**Figure 2:**
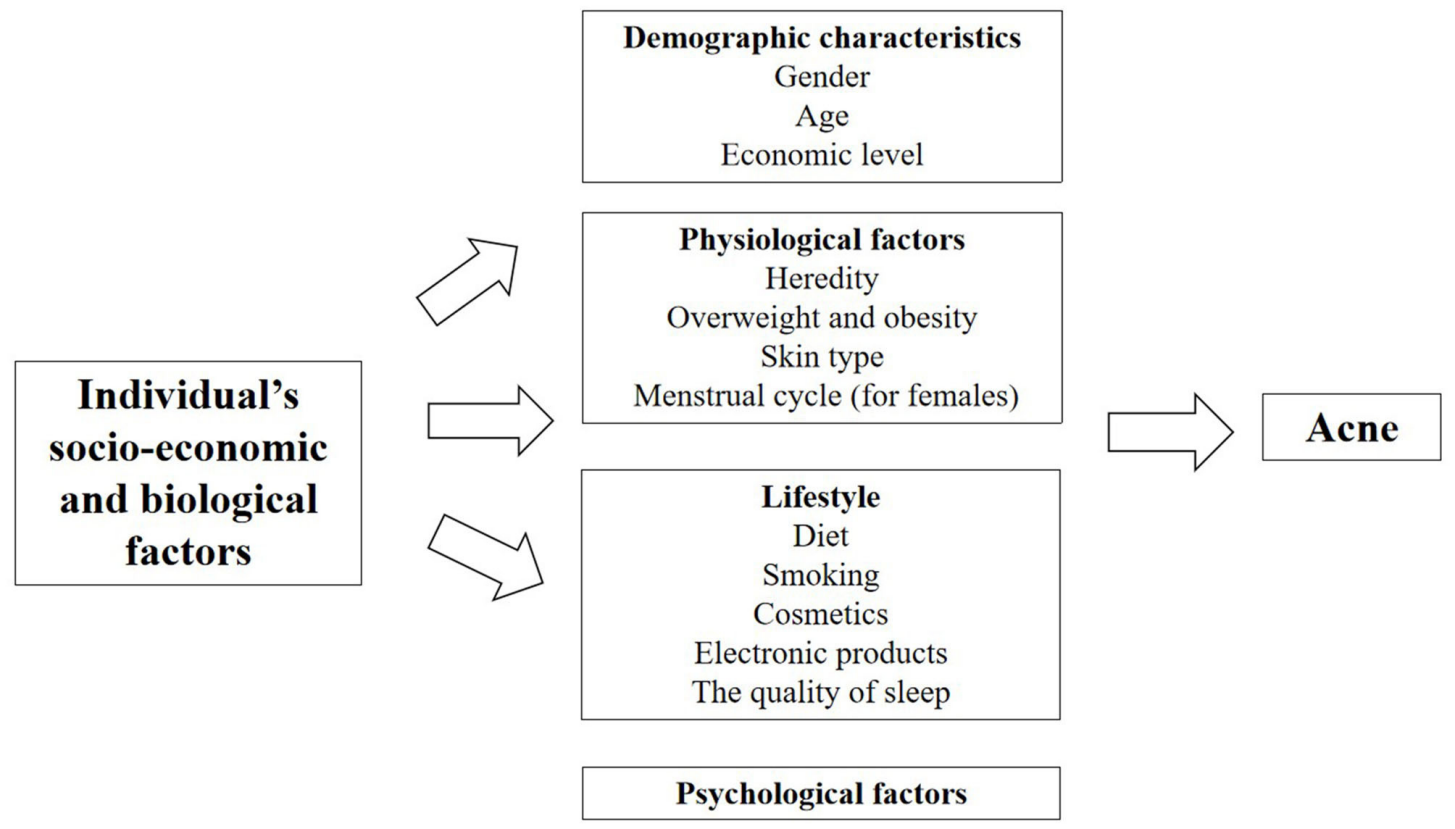
Factors of individual's socio-economic and biological factors affecting acne.

With the rapid urbanization process, there are large-scale migrating and aging populations, changes in dietary structure and lifestyle, and social inequality, leading to a high incidence of chronic diseases (20). As one of the highly recurrent chronic diseases, acne to a large extent is also affected by the relevant demographic and sociological factors, including demographic characteristics, physiological factors, lifestyle and psychological factors (Figure 2, Table 1 in Appendix).

#### Gender

There are differences in endocrine levels between the genders, resulting in differences in the prevalence of acne. The epidemiological survey of acne among undergraduates in the North East China showed that the total prevalence of acne among adolescents was 51.30% (52.74% in males, 49.65% in females) (21). The overall prevalence of acne among European aged 15–24 years was 57.8% (58.28% in males, 56.97% in females) (22). An epidemiology in Singapore showed there were more males than females suffering from adolescence acne (61.3 vs. 38.8%) and more females suffering from post-adolescence acne (69.0 vs. 31.0%) (23). The above studies indicated that more males than females suffered from acne during adolescence and more females than males suffered from acne during post-adolescence.

#### Age

The epidemiology of acne continues to evolve with changes in hormone levels that vary with age. An Italian study of pediatric outpatients aged 9–14 found that 34.3% patients had acne, with the lowest prevalence rate of 6% at age 9, and the incidence of acne increased to 36.3% after the age of 13 (24). From the prevalence of acne among Chinese adolescents, we found increased age was related to higher prevalence and severity of acne vulgaris: 15.6, 44.9, and 70.4% for 10, 13, and 16 years old (25). The European study showed the prevalence of acne was highest at the age of 15–17 and decreased with age (22). These studies confirmed that acne was more common during adolescence.

#### Economic Level

According to family income and regional characteristics, urban residents can be divided into poverty, low-middle income, middle-high income and wealthy groups (26). There were differences in the medical services enjoyed by patients at different economic levels, which might affect the prevalence of acne. According to the Canadian study, only 17% of low-income people earning <$ 20,000 referred a dermatologist, while 24% of high-income people earning more than $ 80,000 consulted a dermatologist (27). Furthermore, there are differences in the prevalence of acne between urban and rural areas. Dreno et al. found acne patients were more likely to live in urban areas with higher socio-economic status (28).

#### Heredity

In clinical work, children of acne patients tend to suffer from acne. Heredity plays a dominant role in the occurrence of acne, especially in severe acne with nodules, cysts and scars. A study of twin models in the UK found that 81% acne variants were caused by genetics and family history, proving that acne have a significant genetic effect (17). An Italian study found that moderate to severe acne is closely related to family history of first-degree relatives (29). An epidemiological study in Iran also showed that the severity of risk of acne increased with the number of family members with acne history, especially a mother with acne history had the greatest impact on acne severity of next generation (30). Studies in China (21) and Europe (22) have found similar results. Other studies demonstrated that the family history of acne was associated with early onset of acne, more skin lesions, and difficult treatment (31). He et al. conducted on a cohort study in the Han population and found that identify two new susceptibility loci at 11p11.2 (damage-specific DNA binding protein 2) and 1q24.2 (selectin L) that are involved in androgen metabolism, inflammation processes and scar formation in severe acne (32).

#### Overweight and Obesity

Obesity has become a global public health crisis. In China, 46% of adults and 15% of children are obese or overweight. There is a significant relationship between the growing prevalence of obesity and chronic diseases (33). Increased secretion of the insulin-like growth factors−1 (IGF-1) in the body (34, 35) and insulin resistance are possible mechanisms by which obesity affects the occurrence of acne (36). In insulin resistance, decreased sensitivity leads to increased insulin release, which in turn leads to increased production of IGF-1 (34). There were studies demonstrated that overweight and obesity (Body Mass Index, BMI ≥ 25 kg/m^2^) were positively correlated with an increased risk of acne (37–39). However, a study in Taiwan, China, indicated that BMI was negatively associated with the number of acne lesions from moderate to severe post-adolescent acne among Taiwanese women aged between 25 and 45 years (40). Recently a nationwide study of 600,404 adolescents indicated overweight and obesity were inversely associated with acne in a dose-dependent manner. In this case, the proportion of adolescents with acne decreased gradually from the underweight to the severely obese group (males, from 19.9 to 13.9%; females, from 16.9 to 11.3%) (41). However, the study had a limitation about the missing information on potential confounders and acne severity. Therefore, the correlation between obesity and acne should be further explored by controlling other influencing factors.

#### Skin Type

Increased sebum production is key factor with interrelated mechanisms, previous study found the sebum level of face was more in population with acne than without acne (42, 43). Excessive sebum secretion is characterized by oily or mixed skin. In addition, Choi indicated the casual sebum level was positively correlated with the number of acne lesions (44). The epidemiology found oily skin and mixed type skin were risk factors to the acne (21, 25).

#### Menstrual Cycle (for Females)

Acne in women is frequently associated with hormonal derangement, including hyperandrogenism. Shrestha et al. showed hormonal alteration in females with adult acne had significant association with irregular menstruation (45). Stoll et al. found 44% of women with acne aggravated in premenstrual period (46). Ghodsi et al. also reported the premenstrual phase was recognized as risk factors for moderate to severe acne (30). In addition, Wei et al. indicated dysmenorrhea was a risk factor to the acne suffers (21). Therefore, dermatologists should consider hormonal alterations in acne patients with irregular menstruation.

#### Diet

The relationship between diet and acne has been a hot topic in the research of acne epidemiology. At present, many studies have confirmed that high sugar diet and dairy products are risk factors for acne (47). Increased sugar intake (≥100 g/d), frequent intake (≥7 times per week) of soft drinks (such as carbonated sodas, sweetened tea drinks and fruit-flavored drinks), and daily consuming dark chocolate were significantly positively associated with acne (30, 48–52). High glycemic load diet can lead to the rise in blood glucose in the body, therefore, islets secrete large amounts of insulin to lower blood glucose, and elevated insulin levels lead to increased secretion of insulin-like growth factors-1 (IGF-1), IGF-1 can increase androgen levels, promote sebum secretion, and promote hyperkeratosis of hair follicle sebaceous glands to affect lipid excretion, thereby inducing or aggravating the occurrence of acne (53–56). And there were studies about a positive association between the incidence of acne and the intake of whole milk and skim milk (57, 58). Milk can also increase the level of IGF-1, which can lead to acne (47, 59–61). In addition, acne can be caused by greasy, fatty foods (62, 63), due to the fact that the release of free fatty acids by triglycerides under the action of P. acnes could promote the development of acne (64). However, it is controversial whether spicy food affects acne. The epidemiological survey of college students in North East China showed that spicy food was a risk factor for acne (21). But other studies have shown that spicy food was not related to the duration or severity of acne (30, 65). Since the two studies did not subdivide the types of spicy foods, the relationship between spicy food and acne needs to be further explored.

#### Smoking

The relationship between acne and smoking remains controversial. The previous studies found that the prevalence of acne was significantly higher in active smokers than ex-smokers or those who had never smoked (7, 66). And the study have also indicated that in contrast to non-smoking group, smokers had significantly higher levels of inflammatory cytokines (67). However, other studies found that people who smoked regularly showed a significantly lower prevalence of severe acne than non-smokers (22, 68, 69). Therefore, the potential influence and mechanisms between acne and smoking need to be further studied.

#### Cosmetics

An improper use of cosmetics may cause the recurrence of acne, the study indicated there is a significant positive correlation between frequent exposure to cosmetics and the severity of acne in adolescent women (70). Studies of Latin America and the Iberian Peninsula have shown consistent results (71). Chinese studies also found cosmetic make-up use was a risk factor of acne (25). The reason was because improper skin care practices (such as essential oils or too oily substrates, makeup, excessive cleansing of the skin and soaps with pH 8.0) can modify skin barrier function and skin sebum areas, especially the microbiome balance, thereby activating innate immunity to trigger inflammation (72).

#### Electronic Products

Visible light emitted by electronic products is a risk factor for acne. Taheri et al. found exposure to short-wavelength visible light emitted from smartphones and tablets could increase the proliferation of Staphylococcus aureus, which could give a rise to an increase incidence of acne (73). Dreno et al. showed people who exposed to screens and tablets before falling asleep were more likely to have acne (28). However, using the computer for <2 h a day was considered a protective factor for acne (21).

#### The Quality of Sleep

Good sleep is essential to good health, poor sleepers [Pittsburg Sleep Quality Index (PSQI) > 5, sleep duration ≤ 5 h] had significantly higher levels of trans epidermal water loss (TEWL) than good sleepers (PSQI ≤ 5, sleep duration 7–9 h). After tape stripping^3^ for 72 h, people with good sleep quality had 30% greater barrier recovery than people with poor sleep. After 24 h of exposure to ultraviolet light, erythema recovery in good sleepers was significantly better (74). When the skin barrier is damaged, the skin's defense system against external stimuli is weakened, which can further lead to skin diseases, especially acne (75). Dreno et al. indicated significantly more individuals with acne than without reported lacking sleep (28). The Chinese study indicate that, sleep duration <8 h per day is a risk factor for acne (21). Surveys in South Korea (76) and Japan (77) have consistent results.

#### Psychological Factors

With the continuous social and economic changes in the contemporary society, the widening of income gap and the increasing stress, the prevalence of mental disorders in China is as high as 9.3% (78). Psychological factors induce the release of neuropeptides and hormones that activate cells to participate in the acne issue (79). The study showed that psychological stress and depression were main risk factors for being acne among college students in the North East China (21). Dreno et al. indicated individuals with acne suffered from significantly higher stress levels than in acne-free individuals (28). Epidemiological surveys in Japan (77), India (80), and South Korea (76) all found that stress was an aggravating factor for the cause of acne.

### Natural Environmental Factors

**Figure 3:**
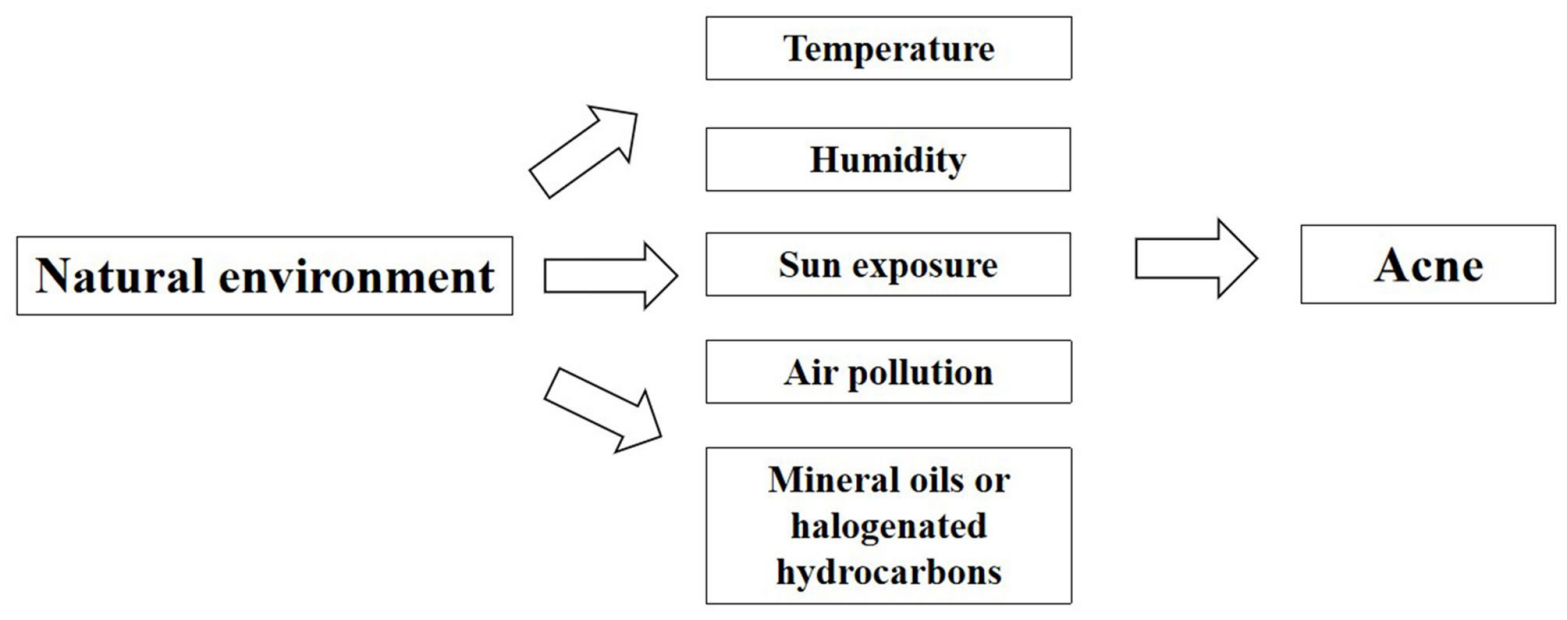
Factors of natural environment affecting acne.

The skin is an important organ that is directly exposed to the external environment. It is also the first barrier against the influence of environmental factors. It protects various tissues and organs in the body from physical, chemical and biological harmful factors. Skin participates in the balance adjustment of the whole body and realize the unification with the external environment. In 2018, Dreno et al. studied the effects of environmental exposure on acne and found that with the changing natural and environmental factors, the response and the susceptibility of body skins to natural environment will accordingly change to a different extent (18). As a consequence of negative impacts on the skin functions, it will increase the occurrence and facilitate the development of acne on people exposed to unfriendly environment (Figure 3, Table 2 in Appendix).

#### Temperature and Humidity

Differences in temperature and humidity in different seasons and regions may lead to different prevalence rates of acne. An Indian study found 82 (47.95%) out of 171 acne patients reported seasonal variations in the severity level of acne. It was statistically significant that there were more aggravated acne issues for acne patients in summer (average temperature 32.2°C, average humidity 49.8%) as compared to rainy (average temperature 31.0°C, average humidity 68.5%) and winter season (average temperature 15.1°C, average humidity 79.7%) (81). The study showed that hot weather was risk factors for acne. However, Dreno et al. found there was no significant difference in prevalence of people with or without acne living in temperate or cold regions. Conversely, acne occurrance was significantly more frequent in hot or humid regions (28). Williams et al. indicated sebum excretion rate varied with local temperature, that is, sebum excretion rate increased by 10% for every 1°C increase in temperature (82). A recent study also showed hot environments cause more production of sebum secretion, especially on the forehead (83). Increased sebum excretion might cause acne to worsening. According to a systematic review and meta-analysis, the prevalence rates of acne in the southern China was higher than that of the northern China, because the southern part is more humid and warmer than the north (84). A study also showed that the higher the altitude, the lower the prevalence of acne, which may be related to higher altitudes and lower temperature and humidity (85). The above studies indicated that hot weather might aggravate acne, but further quantitative studies are needed on the relationship between humidity and acne.

#### Sun Exposure

Sun exposure played a significant role in the incidence of acne. A survey of acne patients in India showed that 26.4% of them developed skin lesions after exposure to sunlight and seasonal variation was observed in 44.5% patients exacerbated, because of increased amount of sunshine exposure in summer months (80). Dreno et al. found acne was significantly more frequent in individuals with moderate or intensive sun exposure due to their work or daily activities (28). Lee et al. showed ultraviolet B irradiation increased the expression of inflammatory cytokines in cultured sebocytes (86).

#### Air Pollution

Air pollution is the most challenging environmental problem for Chinese cities. According to 2016 report on the state of environment in China, only 84 (25%) of the 338 cities have achieved qualified air quality standards for the living of human beings. Over the past decades, people had become more and more concerned about the living condition of urban environment and the health risks related to the increasing and serious air pollution such asPM_2.5_ and PM_10_. Especially, the relevant negative effects of air pollution on the skins have been the key attention of dermatologists and general physicians (87). Clinical studies reported that air pollutants had a deleterious effect on the skin by increasing oxidative stress, leading to severe change of the normal functions of lipids, deoxyribonucleic acid and/or proteins in the human skin (88). Two clinical studies comparing subjects in the highly polluted areas to ones in the less polluted areas in Shanghai and Mexico discovered that skin quality declined with chronic exposure to ambient air pollution (87, 89). A study in Beijing also indicated that increased concentrations of ambient PM_2.5_, PM10, and NO_2_ were positively correlated with numbers of outpatient visits of acne vulgaris over the past 2 years, which further provides an indirect evidence for a link between acne vulgaris and air pollution (90).

#### Mineral Oils or Halogenated Hydrocarbons

Chloracne is also known as occupational acne, it is a special type of acne caused by exposure to mineral oils or certain halogenated hydrocarbons in production labor (91). The increase of cysts in number is a signal of aggravation of chloracne (92). Dreno et al. found the vast majority of people with acne were significantly more exposed to tar, solvent emanation and crude oil or oil emanation than people without acne (28). Therefore, an effective way to prevent chloracne is to avoid the contact with halogenated hydrocarbons.

### Social Environmental Factors

**Figure 4:**
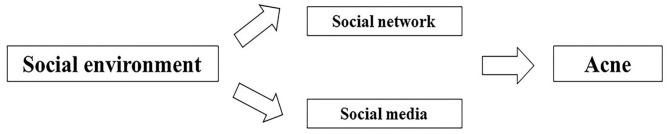
Factors of social environment affecting acne.

In addition to natural environment, social environment plays a critical role in the health, behavioral norm and social adaptation of the population as a whole (Figure 4, Table 3 in Appendix).

#### Social Network

Social network refers to the relatively stable relationship system established by the interaction between different individuals comprising the society, with individuals embedded in the thick webs of social relationships and interactions (93). Recently, there has been much emphasis on the role that social network plays in our physical health, mental health, social behavior and social adaptation (94). According to Cohen-Cole et al. found a friend's acne problems increased an individual's odds of having acne problems (95), which is potentially associated with the similar and interactional living environment, lifestyle and diet among friends. In addition, social networks may have an indirect impact on the occurrence of acne. In recent studies, it has indicated that obesity could spread through various social relationships, which means that the chance of a person developing obesity increases if his or her friend, sibling or spouse becomes obese, suggesting that people embedded in social networks are subject to the influence from the evident appearance and behaviors of those around them (96, 97). Moreover, there are a growing number of studies demonstrating that social networks could exert impacts on the psychology and behavior, such as depression, anxiety, smoking, drinking and aggression (98–100). In previous studies, obesity, anxiety, depression, and smoking have been identified as the risk factors for acne (21, 28, 37, 66). Therefore, it is possible that social networks have impacts on obesity, anxiety, depression, and smoking among peers, thus impacting on acne indirectly. In this regard, if more studies can be conducted to determine the mechanism of social network behind the occurrence and development of acne, the effective means of behavior intervention can be developed in the future.

#### Social Media

With the rapid advancement of novel technology, social media has made it convenient for patients to communicate their skin diseases, share treatment and skin care, and even get access to the education on their illness. As revealed by Yousaf et al., 45% of the patients resorted to social media for expert advice on acne treatment (54% of women vs. 31% of men), which evidences the influence of social media on acne treatment. Nevertheless, merely 31% of the participants turning to social media made the changes fully compliant with the American Academy of Dermatology (AAD) clinical guidelines (101). According to Borba et al., the videos of acne education that viewers seek online are clearly inaccurate and poor in quality (102). The incorrect or irregular treatment suggested on social media may contribute to the aggravation of acne. Therefore, the dermatologist appointment on social media is expected to provide the right information to help educate patients.

### Built Environmental Factors

The built environment is human-made or modified surroundings, such as buildings, land use (e.g., layout of communities, transportation systems, infrastructures), or green space (12). Research has indicated that built environments and health issues are inextricably linked, because exposure factors affects body condition of human beings (103). Patterns of land development, transportation infrastructure, and building location and design—the built environment could affect the natural environment by replacing natural areas and changing functions and services of ecosystem, which are closely related to the exposure of human beings in the environment.

At present, a growing number of studies have focused on the impact of built environment on health (Table 4 in Appendix), especially those chronic diseases such as obesity (13), cardiovascular disease (15) and mental health (14). Studies indicated that obesity was positively associated with population density and the availability of fast-food outlets from the people's residence (104–108). Moreover, other studies also found the incidence of cardiovascular disease was significantly higher with more fast-food outlets than areas with no fast-food outlets (15, 109). On the contrary, compared with cities with less green space, cities with larger or medium green areas had a lower risk of suicide (14, 110). Green plants affect people's psychological function, making them less susceptible to stressful life events, that is, alleviating stress and supporting their reflection on life (111). In addition, the diversity of resources, ease of access, mobility, personal safety, and street connectivity were closely associated with the higher mental well-being scores among the neighborhoods (112). Active transport, including walking, cycling and the use of public transport, delivered health benefits in reducing type 2 diabetes and the mortality due to various causes (113).

**Figure 5:**
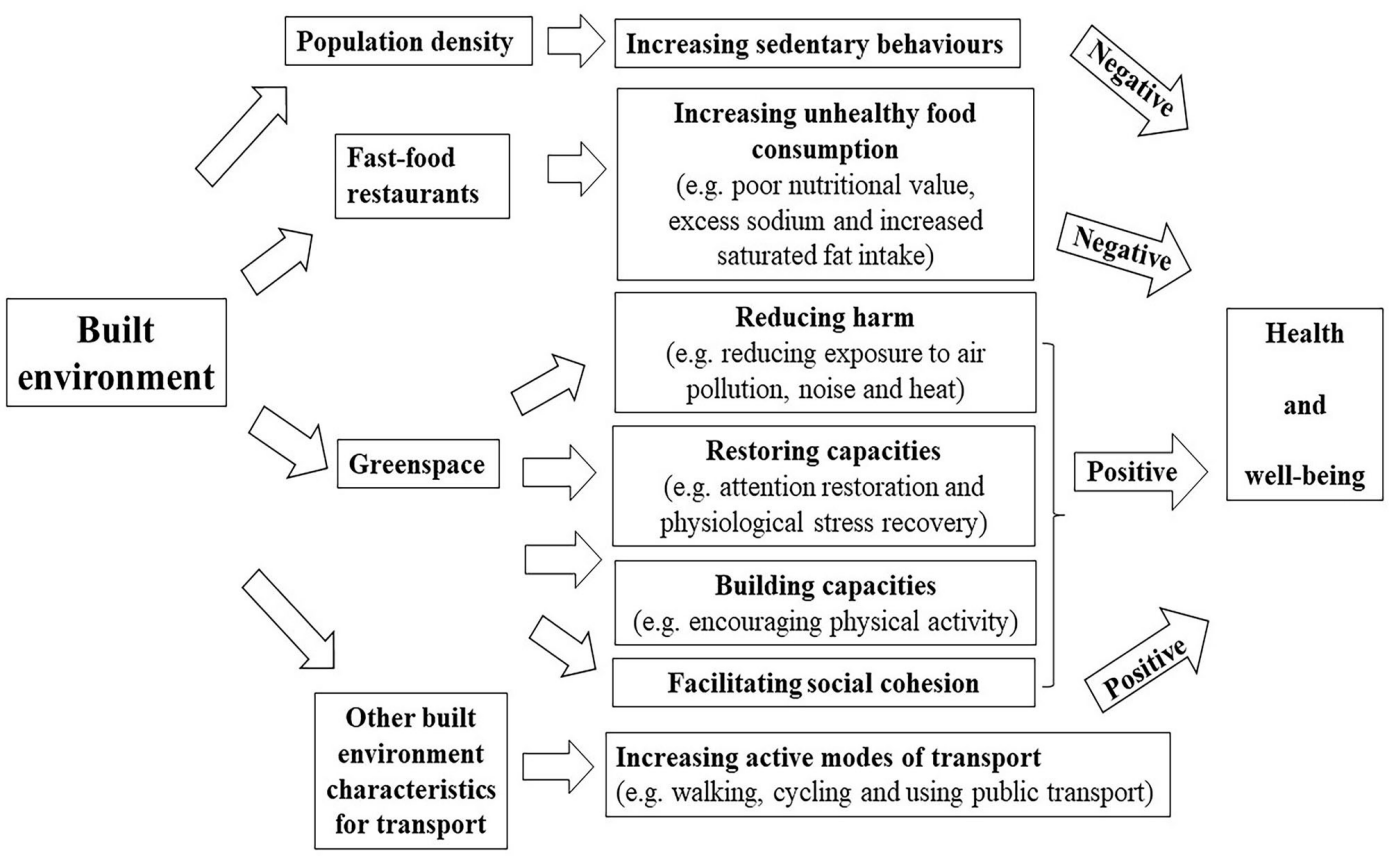
Pathways linking built environment (fast food restaurant and greenspace) to health outcomes (negative: there is a negative effect; positive: there is a positive effect).

All these studies indicated that the built environment played an important role in the incidence of chronic diseases. In the existing research, it has confirmed that built environment exerted some indirect impacts on the health of individuals. Population density, fast food outlets, green spaces and public transport accessibility are exemplified as shown in Figure 5. With regard to population density, the potential mechanism lying behind the correlation between residential density and overweight may be associated with sedentary behaviors, as indicated by Xu et al. who demonstrated that the participants in higher-density areas spent more time in sedentary behaviors than those in lower-density areas (114). In terms of fast food, on the one hand, fast food outlets jeopardize the health through high-density fast food restaurants, increasing the chances of eating unhealthy food, frequent fast-food consumption further leads to low nutritional value, excessive sodium intake, increased saturated fat intake, which is linked to cardiovascular disorders, obesity and other metabolic diseases (115–118). While green spaces promote health through four general pathways (119, 120). The first pathway is reducing harm (e.g., reducing exposure to air pollution, noise and heat). With increasing outdoor levels of certain greenspace indicators, indoor levels of PM2.5 and noise annoyance are reduced (121, 122). The second pathway is restoring capacities (e.g., attention restoration and physiological stress recovery). Viewing plants and other natural environmental features can evoke positive emotions very quickly, thereby shielding negative thoughts and emotions, improving or turning off stress responses (111). The third pathway is building capacities (e.g., encouraging physical activity). Green spaces may provide a safe, accessible and attractive environment for physical activity (123). The fourth way is to promote social cohesion. Green spaces provide an environment for contact with neighbors, which may increase social cohesion within the community (124). In respect of other built environment characteristics for transport, there is evidence that people using public transport are four times more likely to reach the recommended amount of physical activity than ordinary motorists, which is equivalent to an additional 33 min of walking per day (125), moreover, active travel, particularly walking and cycling, has been recommended because of the health benefits associated with increased physical activity (126). Increased physical activity is associated with lower body weight (127).

Skin is one of the main interfaces between human body and external environment and is one of the main barriers to prevent pathogens to invade human body. The main function of the skin is to act as a physical barrier to protect our bodies from potential attack by foreign organisms, toxins, or any other external physical, chemical, or organic factors (128). The built environment may affect the skin through the following mechanisms. Firstly, high population density environment, as a psychosocial stress, induced the impairment of barrier function and water retention property concomitant with decline of ceramide and pyrrolidone carboxylic acid in the stratum corneum (129). Secondly, Yamane et al. suggested high-fat diet reduces the levels of type I tropocollagen and hyaluronan in the skin by inhibiting the effects of transforming growth factor (TGF)-β1, IGF-I and adiponectin, and these effects are harmful for skin function (130). In addition, Meeran et al. showed high-fat diet might increase susceptibility to inflammation-related skin diseases, including the risk of skin cancer (131). So frequent fast-food consumption with high-fat may have a negative impact on the skin. Thirdly, there is increasing evidence that air pollution (e.g., PM_2.5_, PM_10_, NO_2_, SO_2_) exerts negative effects on the human skin, it may activate cell metabolism and inflammation (132). Moreover, it has been reported that PM is associated with increased risks of skin diseases, especially skin aging (133), acne (87), atopic dermatitis or eczema (134). Through the above analysis on mechanism, greenspace can reduce exposure to air pollution. Accordingly, the reduction of pollutants is a protective factor for the skin, and the occurrence of skin diseases may also be reduced. Fourthly, psychosocial stress has a negative impact on skin disease by activating the expression of inflammatory cytokines or compromising both permeability barrier homeostasis and stratum corneum integrity (135, 136). Thus, greenspace may protect the skin by reducing psychological stress. Lastly, the study have found high physical activity group showed a positive outcome with respect to wrinkles compared to low and middle physical activity group (137). Therefore, we can guess that greenspace, better public transport accessibility and active travel (e.g., walking and cycling) may be beneficial for reducing wrinkles by providing more space for increasing physical activity.

Acne is a common and chronic inflammatory skin disease, Dreno et al. confirm that internal and external exposome factors had a significant impact on acne (28). Thus, the built environment is closely related to our lives and may also have a potential impact on acne.

## Discussion

### Building the Indirect Relationship Between the Built Environment and Acne

**Figure 6:**
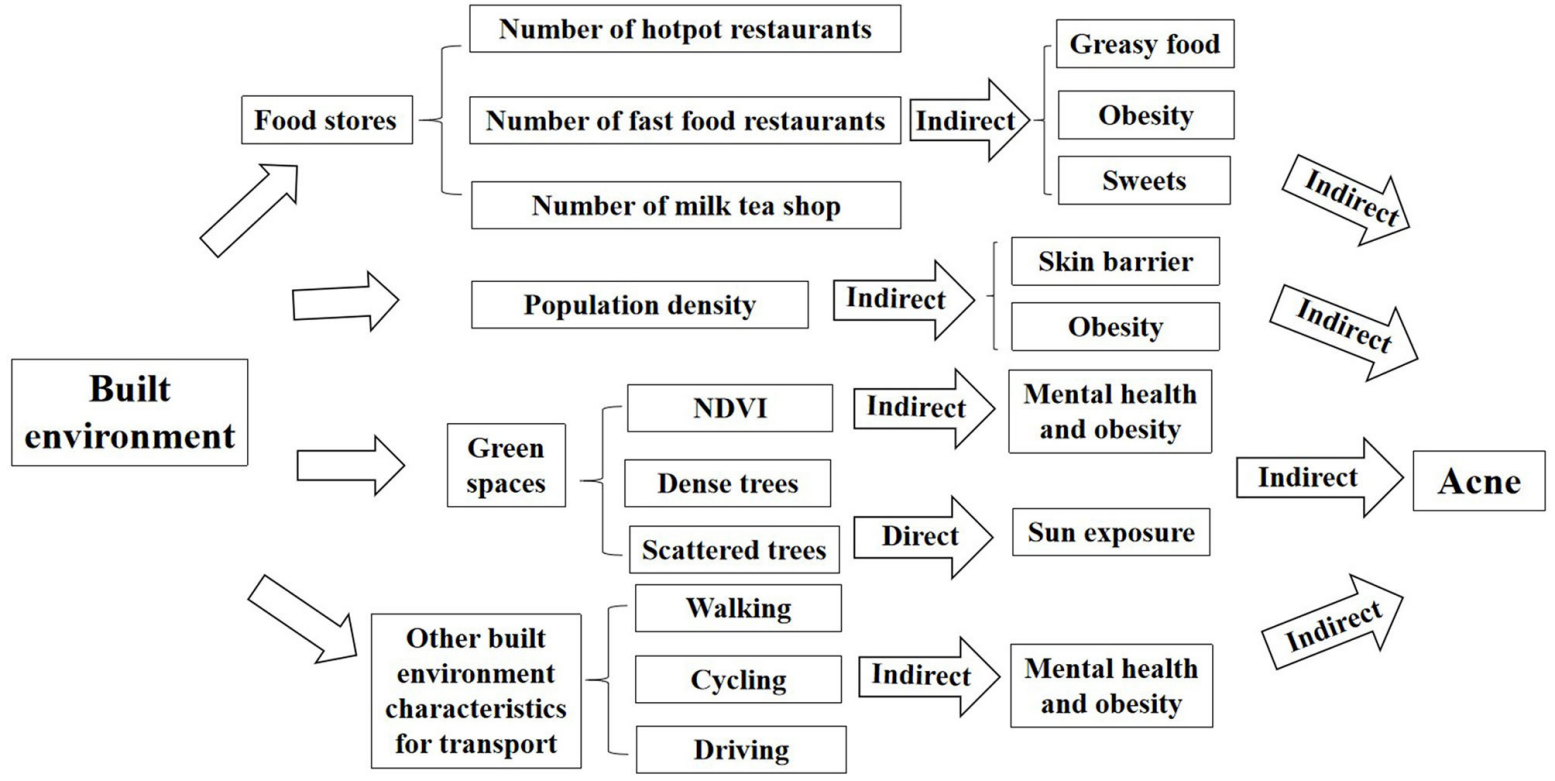
The framework of impacts of built environment on acne (indirect: there is an indirect effect; direct: there is a direct effect).

There is still a lack of scientific research on whether the built environment is related to the occurrence of acne. To bridge this knowledge gap, we will explore the indirect relationship between the built environment and acne, and provide a scientific basis for future epidemiological investigations (Figure 6).

#### Population Density

Population density refers to the average number of people living on land per unit area. According to the study of Tsinghua–Lancet commission on healthy cities in China, between 1978 and 2015, China experienced the largest population migration from rural areas to cities in human history. The urbanization rate increased from 17.9 to 56.1%, and the urban population also increased from 170 to 771 million (1). An epidemiological survey in Asia has found an increase in the prevalence of diseases in areas with high population density (138). Xu et al. indicated residential density was positively associated with being overweight among urban Chinese adolescents (114). High density can increase obesity, which may lead to acne. In addition, an animal study found that high population density in mice damaged skin barrier function and TEWL (129). When the skin barrier was damaged, the skin defense against external exposure was weakened, which could lead to acne (75). However, whether population density affects acne is unknown, the correlation between population density and acne needs further study.

#### Food Stores

On the one hand, in summary of the factors affecting acne, we found that greasy food, spicy food, dairy products, and sweets are risk factors for acne. So hotpot restaurants and milk tea shops around residents' residences may indirectly affect acne, because a higher number of hotpot restaurants and milk tea shops around the location people work and live, it may be more likely to provide greasy food, spicy food, dairy products, and sweets to people, which could largely increase the possibility of people being acne. On the other hand, obesity is a risk factor for acne, which implies that types and numbers of restaurants around the residential and work location of people could be related to their obesity. A number of studies in the US (139), New York (105), Utah (106), the UK (13, 140), Porto (104), New Orleans (141), and China (142) showed higher fast food restaurant density was significantly associated with higher obesity rates among students. Therefore, an increased number of fast food restaurants near the address may increase the risk of obesity, which may indirectly affect the occurrence of acne. The number of fast food restaurants near the address is one of the measurement indexes of built environment, but whether it affects acne, needs empirical studies to verify.

#### Green Spaces

Green spaces encourage people to spend more outdoor time for sports, entertainment and social activities. It could have unexpected but important consequences for health in countries with very high levels of ultraviolet (UV) radiation, because sun exposure is one of the risk factors for acne (28). A study in Australia showed that compared to people with 0–20% green space, those with 80% green space had a 9% higher chance for skin cancer. Because people who live near green spaces have higher exposure to outdoor environments and the incidence of skin cancer increases accordingly (143). Different types of green spaces may affect acne, for instance dense trees could reduce UV radiation and therefore protect skin by providing shade, while scattered trees are less protective of skin because they cannot block UV radiation (144). Moreover, there is growing evidence that natural outdoor environments, such as green spaces (i.e., grass, forests, or parks) was increasingly shown to promote mental health. A study in the Netherlands found cities with a large proportion of green space (>85%) or a moderate proportion of green space (>25% to ≤85%) had a lower suicide risk than cities with less green space (≤25%) (14). Epidemiology research have confirmed that depression and stress are important factors of acne, which means that green space may indirectly affect the occurrence of acne by affecting mental health. According to a study in South Africa, each participant was assigned a value for a green living space, which was obtained from a normalized difference vegetation index (NDVI) generated by a satellite based on the global positioning system (GPS) coordinates of their household location (110). Liu et al. suggested sufficient green infrastructure at the neighborhood scale could protect against depression and promote mental well-being in Chinese urban settings (145–150). Furthermore, green space may be associated with decreased risk of excess weight/obesity (151–153). Therefore, an increase in the proportion of green space near the living and working places may encourage people to participate in more outdoor activities so that decrease the risk of stress, depression and obesity, which may indirectly decrease the occurrence of acne. Green space is one of the measurement indexes of built environment, but its effect on skin, especially acne, needs to be verified by empirical studies.

#### Other Built Environment Characteristics for Transport

Land-use diversity and street connectivity can influence the choice over transport mode, which in turn affects health (154). In previous studies, it has been demonstrated that a reduction to the distances to public transport could reduce motorization, which means a modal shift from private motorized vehicles to walking, cycling, and public transport can help improve physical health for all urban residents, for example, reducing obesity, diabetes, cardiovascular disease and respiratory diseases (155–157). Then, the characteristics of built environment for transport may also have impacts on acne indirectly. On the one hand, improving accessibility to public transport will improve population health by promoting the engagement in physical activities (158). A European study was conducted to demonstrate that BMI dropped when people started or increased cycling, but increased when car was used, suggesting the health benefits were created by active mobility (159). Those preferring walking or cycling exhibited a lower BMI over time than those using cars on a long-term basis (160). Additionally, Liao et al. found out that Taiwanese adults mainly reliant on public transport for travel showed a higher likelihood of engagement in transport-related physical activity and a lower level of risk of developing obesity than those who traveled by walking, cycling, or private vehicles (161). On the other hand, different transport modes had different impacts on mental health. Cycling and walking were linked to the positive self-perception about health (162). However, the commute by car has been associated with high stress and lower mental well-being (163, 164). In general, people walking or cycling as a frequent means of commute may have lower BMI and better mental state, which may contribute indirectly to reducing the occurrence of acne, regarding that obesity, psychological stress and depression are the risk factors for acne. Thus, reducing obesity and improving mental health may help reduce the incidence of acne. While the commute by car might produce the opposite result. However, the correlation between the characteristics of built environment for transport and acne may also be influenced by other potential factors, for example, walking without the protection against solar radiation may increase sun exposure, which will aggravate acne as well. Therefore, further research is required to confirm the potential relationship between them.

## Strengths and Limitations

The core merit of our paper lies in a thorough review of the relevant socio-economic, biological and environmental factors that could impact on acne as well as of the underlying mechanisms. Based on that, the direct and indirect relationships were established between built environmental factors (population density, food stores, social network, transport mode, land use) and acne by reviewing the effects of built environment on health, thus providing a theoretical basis for a further research on the effective means of behavior intervention.

It is essential to acknowledge the weaknesses of the current study, that is, there remain few studies focusing on the impact of built environment on acne. In this situation, attempt was made in our study to determine the indirect relationship between built environment and acne by exploring its impact on obesity and psychology. It was possible to miss some information about the potential confounders impacts on acne.

## Conclusions

Acne refers to a polygenic genetic disorder affected by the interaction between genetic and environmental factors. In this study, it was concluded that acne mostly occurs during adolescence, with age and gender playing a significant role in its occurrence. Besides, the prevalence of acne showed a decreasing trend with age. Males outnumbered females in terms of adolescence acne while it was the opposite in terms of post-adolescence acne. Moreover, acne can be affected negatively by such influencing factors such as family history, overweight, obesity, oily and mixed skin, irregular menstrual cycles, sweet food, greasy food, dairy products, smoking, the improper use of cosmetics, the long-term use of electronics, the poor quality of sleep and stress. In addition, environmental factors play a crucial role, along with various natural environmental factors, including temperature, sun exposure, air pollution, mineral oils and halogenated hydrocarbons, serve as risk factors for acne. Moreover, a further qualitative research is required to figure out the impact of humidity on acne. Lastly, social networks and social media can affect acne as well.

However, the impact of built environment on acne has yet to be reported in previous studies. Thus, an attempt was made in this study to determine the indirect relationships between built environment and acne regarding the impacts of built environment on the risk factors for acne. To cure such a chronic disease, it is necessary to understand the indirect relationship between the built environment and acne by gaining understanding as to the impact of traditional factors on the pathogenesis of acne.

In the future, the study conducted from the perspectives of medicine, sociology of population and geography will be required, and more empirical studies are required to reveal the specific relationship between built environment and acne. The potential built environmental factors for acne ought to be analyzed by collecting the data on demographic characteristics, physiological factors, lifestyle, psychological factors, as well as population density, food stores, green space, climate, pollution status, and so on in relation to the residence of patients with acne, in combination with traditional research factors. In doing so, the impact of built environmental factors on acne can be fully understood to provide specific guidance on reducing the prevalence of acne.

## Author Contributions

JY, HY, and LH were equally responsible for writing, editing, and literature review. All authors were involved in manuscript preparation, approved the final version, and agreed to be accountable for all aspects of the work.

## Conflict of Interest

The authors declare that the research was conducted in the absence of any commercial or financial relationships that could be construed as a potential conflict of interest.

## Footnotes

^1^Spironolactone is $350, oral antibiotics are $501, topical antibiotics are $920, topical retinoids are $1,805, topical combination antibiotics are $2,282, topical combination antibiotics and retinoids are $3,770, the median cost for isotretinoin in males and females are $3,227 and $3,806 respectively.

^2^If the topic of studies was not only associated with acne but also other diseases, there might exist confounding effects issues in between.

^3^Applying tape to disrupt the skin barrier.
