# Supplementary material for: A Review of Advancement on Influencing Factors of Acne: An Emphasis on Environment Characteristics
Source: Front Public Health. 2020 Sep 17;8:450. doi: 10.3389/fpubh.2020.00450 (PMC7527424; doi:10.3389/fpubh.2020.00450)
Supplement: Supplementary file 1 [file Table_1.DOCX]

**Table 1 Summary of selected studies between acne and sociology of population**

| **Author and year** | **Location** | **Aim of the study** | **Sample** | **Variables of the study** | **Statistical method** | **Main results** |
| --- | --- | --- | --- | --- | --- | --- |
| Napolitano et al. (2018) | Italy | To investigate the prevalence of acne among 9 to 14-year-old patients. | N=693 children | Demographic characteristics,  Past personal history of acne | Fisher’s exact test | 34.3% of pediatric outpatients had acne, and the prevalence of acne increased with age, with the highest prevalence at age 13 (36.3%) and the lowest prevalence at age 9 (6%). |
| Wei et al. (2010) | North East China | To investigate the epidemiological features of adolescent acne in North East China and identify the impact of acne. | N=5696 undergraduates | Demographic characteristics,  Socioeconomic condition,  Acne status,  Familial hereditary history | Chi-squared test | The overall prevalence of acne among adolescents was 51.30% (52.74% in men and 49.65% in women).  Risk factors: family history, mental stress, menstrual disorders, frequent insomnia, being men, dysmenorrhea, anxiety, sleep <8 hours per day, depression, fried foods, study stress, high-fat diets, spicy foods, oily skin, mixed skin.  Protective factors: dry skin, neutral skin, frequent fruit consumption and computer use <2 hours per day. |
| Han et al. (2016) | Singapore | To compare epidemiological data of adolescence (<25 years) and post-adolescence (≥25 years) acne patients diagnosed between 2004 and 2013. | In 2004, N=4447  In 2013, N=5723 | Demographic characteristics,  Clinical characteristics | Chi-squared test | There were more males than females with adolescence acne (61.3% vs. 38.8%) and more females with post-adolescence acne (69.0% vs. 31.0%). |
| Bataille et al. (2002) | the UK | To explore the influence of genetic and environmental factors on acne. | N=458 pairs of monozygotic and 1099 pairs of dizygotic twins | Demographic characteristics,  Family history | Chi-squared test,  Correlation statistics | 81% acne variants were caused by genetic factors and family history, proving that acne have a significant genetic effect. |
| Di Landro et al. (2012) | Italy | To assess the impact of family history, personal habits, dietary factors, and menstrual history on diagnosis of moderate to severe acne. | N=563 | Demographic characteristics,  Family history,  Personal habits,  Dietary factors,  Menstrual history | Chi-squared test,  Logistic regression | Family history, body mass index, and diet may influence the risk of moderate to severe acne. |
| Ghodsi et al. (2009) | Iran | To identify the prevalence, severity and affecting factors of acne vulgaris among adolescents. | N=1002 | Demographic characteristics,  Family acne history,  Personal habits,  Emotional stress,  Menstruation | Chi-squared test,  Logistic regression | The risk of acne severity increased with the number of family members who had a history of acne. Mother with acne had the greatest effect on the severity of acne. Increased age, sebum secretion, premenstrual period, stress, sweets and greasy foods were risk factors for moderate to severe acne. In contrast, gender, spicy food, and smoking were not related to the severity of acne. |
| Ballanger et al. (2006) | France | To compare clinical and evolutive features of acne and response to treatment in patients with acne with or without family history of acne. | N=151 | Demographic characteristics,  Family acne history | Chi-squared test | The family history of acne was associated with early onset of acne, more skin lesions, and difficulty of treatment. |
| Burris et al. (2014) | New York | To examine the differences in dietary factors between groups of self-reported acne severity. | N=248 | Demographic characteristics,  Self-reported acne severity,  Dietary factors | Chi-squared test | Participants with moderate to severe acne reported greater dietary glycemic index, added sugar, total sugar, number of milk servings per day, saturated fat, and trans-fatty acids, and fewer servings of fish per day than those with no or mild acne. 58.1% participants thought diet aggravated or affected acne. |
| Huang et al. (2019) | China | To investigate the association of soft drink consumption and the intake of sugar from soft drinks with the prevalence of acne in adolescents. | N=8226 | Demographic characteristics,  The intake of soft drinks | Chi-squared test,  Two-level logistic models,  Generalized additive models | Daily consumption of soft drinks significantly increased the risk of moderate to severe acne in adolescents, especially when the sugar intake from any type of soft drink exceeds 100 g per day. |
| Aksu et al. (2011) | Eskisehir, Turkey | To identify the prevalence and factors of acne in adolescents in the city of Eskisehir, Turkey. | N=2300 | Demographic characteristics,  Dietary habits | Mann–Whitney U and Kruskal–Wallis H tests,  Chi-squared test,  Pearson correlation | Acne prevalence was 60.7%. Frequent fat intake, frequent sugar intake, frequent eating sausages, burgers, frequent eating pastries, cakes were associated with increased risk for acne. |
| Adebamowo et al. (2004) | the United States | To evaluate the relationship between intakes of dairy foods and acne during high school. | N=47,355 | Demographic characteristics,  Dietary habits | Wilcoxon rank sum test,  Chi-sqaure test | Consumption of whole and skim milk was positively correlated with acne |
| Aalemi et al. (2019) | Kabul, Afghanistan | To investigate the association of dairy intake and acne in Kabul citizens. | N=558 | Demographic characteristics,  Dietary habits | Logistic  Regression Analysis | Dairy intake may be a factor contributing to acne. |
| Nguyen et al. (2016) | the United States | To explore the link between diet and acne. | N=50 | Demographic characteristics,  Dietary habits |  | Fried/greasy foods and chocolate may serve as acne triggers. |
| Abo El-Fetoh et al. (2016) | Arar, Kingdom of Saudi Arabia | To estimate the prevalence of acne vulgaris, to describe the sociodemographic characteristics of cases, and to determine the aggravating factors and the psychological impact of acne vulgaris. | N=400 | Demographic characteristics,  Family history,  Dietary habits,  Smoking,  seasonal variation | Chi-sqaure test,  t-test | The overall prevalence of acne was 53.5%. Family history, skin cleanliness, certain diets (fatty foods, chocolate, spicy foods, cola beverages) and smoking were risk factors for acne. There was clear seasonal variation, with a higher prevalence mainly in summer. |
| El Darouti et al. (2016) | Egypt | To assess the relationship between the dietary intake of salty and spicy food and the onset, severity, duration of acne. | N=200 | Demographic characteristics,  Dietary assessment | Multiple comparison post-hoc test,  nonparametrical Mann–Whitney test,  Spearman test | Neither salty nor spicy food correlated with duration or severity of acne. |
| Schäfer et al. (2001) | Germany | To identify the prevalence and demographic factors of acne. | N=896 | Demographic characteristics,  Medical history,  Alcohol and cigarette consumption | Multiple logistic regression analyses | The prevalence of acne was significantly higher in active smokers than ex-smokers or those who had never smoked. |
| Klaz et al. (2006) | Israel | To determine the relationship between smoking and acne. | N=27083 | Demographic characteristics,  Daily cigarette smoking | Multiple logistic regression model | Active smokers showed a significantly lower prevalence of severe acne than nonsmokers. |
| Wolkenstein et al. (2015) | Switzerland | To determine the relationship between dietary factors, smoking and acne | N = 1375 | Demographic characteristics,  Dietary factors,  Cigarette consumption | Multiple logistic regression analysis | Chocolate, sweets and cannabis smoking were associated with acne. |
| Wolkenstein et al. (2018) | Belgium, Czech and Slovak Republics, France, Italy, Poland and Spain | To determine the prevalence of self-reported acne among young people in Europe and evaluate the effect of lifestyle on acne. | N = 10521 | Demographic characteristics,  Lifestyle (e.g. dietary habits, use of tobacco, cannabis and alcohol),  family history | Logistic regression analysis | The overall prevalence of self-reported acne was 57.8%. Acne history of parents was associated with an increased probability of having acne. Increasing age and smoking tobacco were associated with a reduced probability of acne. |
| Perera et al. (2018) | Colombo, Sri Lanka | To analyze the association between frequent exposure to cosmetics and severity of acne in adolescent females. | N=140 | Demographic characteristics,  The patterns of cosmetic usage | Pearson correlation coefficient value,  Chi-square value | Frequent exposure to cosmetics had a positive association with the severity of acne in adolescent females. |
| Kaminsky et al. (2019) | Latin America and the Iberian Peninsula | To identify the risk factors for acne. | N=1384 | Demographic characteristics,  Family history,  Endocrine and metabolic disorders,  Menstrual history,  Personal habits,  Treatments for acne | Multiple logistic  regression | The male gender, use of cosmetics, age of onset of adolescence, and signs of hyperandrogenism were associated with the severity of acne. |
| Dreno et al.  (2019) | France, Germany, Italy, Brazil, Canada and Russia | To assess the most involved exposure factors in acne. | N=6679 | Demographic characteristics,  Nutrition and nutritional supplements,  Occupational factors,  Medications,  Pollutants,  Psychosocial and modern lifestyle factors,  Weather conditions | Multiple logistic regression analysis | Nutrition, pollution, stress and harsh skin care, as well as climate and sun exposure may be considered the most frequent factors associated with acne. |
| Suh et al. (2011) | Korea | To analyze the incidence of acne and to identify its demographic and clinical features and aggravating factors. | N=1236 | Demographic characteristics,  Lifestyle | Chi-squared test,  t-test | Stress, lack of sleep, smoking, alcohol consumption, and menstruation aggravated acne. |
| Kubota et al. (2010) | Japan | To assess the prevalence of acne, knowledge about acne, self-management of acne and emotional well-being. | N=1443 | Demographic characteristics,  The Mental Health Inventory (MHI) subscale of the Short Form 36 | Wilcoxon signed-rank test,  t-test | Students with acne were significantly more depressed than those without acne and female students were significantly more depressed than male students. |
| George et al.  (2018) | India | To analyze various factors that aggravate acne in Indian adults. | N=110 | Demographic characteristics,  Personal habits | Chi-squared test | Food, cosmetics, stress, sun exposure, first degree relatives with present or past history of acne, premenstrual and seasonal variation were aggravating factors of acne. |
| Halvorsen et al. (2012) | Oslo, Norway | To demonstrate the possible relationship between BMI and acne in adolescents. | N=4744 | Demographic characteristics,  BMI | Regression analysis | Overweight and obesity were associated with acne in girls aged 18 and 19, but the same relationship was not observed in boys. |
| CESKO et al. (2009) | Essen, Germany | To investigate factors affecting acne. | N=100 | Demographic characteristics,  BMI,  Smoking behavior | Chi-squared test | Smoking and obesity might be related factors to acne. |
| Karciauskiene et al. (2014) | Lithuania | To estimate the prevalence of acne among schoolchildren and its association with puberty, BMI, acne history of parents, nutritional habits, smoking and alcohol consumption. | N=1277 | Demographic characteristics,  BMI,  Nutritional habits,  Smoking and alcohol consumption | Binary logistic regression | The overall prevalence of acne among schoolchildren was high and age-dependent. Overweight/obesity and a history of acne in both parents were risk factors for acne. |
| Lu et al. (2015) | Taiwan | To demonstrate the relationship between BMI and acne lesion counts in women with post-adolescent acne. | N=104 | Demographic characteristics,  BMI | Multiple linear regression analysis | BMI was negatively associated with the number of acne lesions from moderate to severe post-adolescent acne among Taiwanese women. |
| Snast et al. (2019) | Jerusalem, Israel | To determine the association between BMI and acne in youths. | N=600404 | Demographic characteristics,  BMI | Logistic regression | In youths, overweight and obesity were inversely associated with acne in a dose-dependent manner. |
| Haider et al. (2005) | Ontario, Canada | To determine whether socioeconomic status influences access to specialist care by a dermatologist for the management of acne and whether the urban-rural dwelling status of patients affects access to specialist care. | N= 295,469 | Demographic characteristics,  Annual neighborhood household income | Chi-squared test | Only 17% of low-income people earning less than $ 20,000 turned to a dermatologist, while 24% of high-income people earning more than $ 80,000 consulted a dermatologist |
