# Supplementary material for: A Review of Advancement on Influencing Factors of Acne: An Emphasis on Environment Characteristics
Source: Front Public Health. 2020 Sep 17;8:450. doi: 10.3389/fpubh.2020.00450 (PMC7527424; doi:10.3389/fpubh.2020.00450)
Supplement: Supplementary file 2 [file Table_2.DOCX]

**Table 2 Summary of selected studies between acne and natural environmental factors**

| **Author and year** | **Location** | **Aim of the study** | **Sample** | **Variables of the study** | **Statistical method** | **Main results** |
| --- | --- | --- | --- | --- | --- | --- |
| Narang et al. (2018) | India | To assess the seasonal variation of acne in patients and to correlate it with  the monthly temperature and humidity over a period of 1 year. | N=171 | Demographic characteristics,  Seasonal changes in acne,  The mean temperature and humidity of each month | Z test,  One‐way analysis of  variance (ANOVA) | Both temperature and humidity play an important role in the pathogenesis of acne and also contribute to the recurrence of acne. The aggravation of acne usually occurred in summer and rainy season. |
| Li et al. (2017) | China | To assess the prevalence of acne in mainland China and quantify its relationship with gender, region and age. |  | Demographic characteristics | Meta-analysis | The prevalence of acne in south China was higher than that in north China. |
| Robeva et al (2013) | Sofia, Bulgaria | To investigate the interrelations between the presence of acne and several variables associated with somatic growth, pubertal maturation, and environmental conditions (altitude and regions of residence) | N=6200 | Demographic characteristics,  The altitude of residence | Binary logistic regression | The acne frequency decreased with the increasing of the altitude where the boys lived. |
| George et al.  (2018) | India | To analyze the various factors that aggravate or precipitate acne vulgaris in Indian adults. | N=110 | Demographic characteristics,  Personal habits | Chi-squared test | Sun exposure was aggravating factors of acne. |
| Lefebvre et al. (2015) | Mexico | To evaluate the effect of pollution on skin. | N=189 | Two different districts with different levels of pollution,  Biochemical and clinical skin parameters | t-test,  Mann-Whitney test | Polluted environment had a negative effect on skin quality. |
| Lefebvre et al. (2016) | Shanghai, China | To evaluate the effect of urban pollution on skin. | N=159 | Two different districts with different levels of pollution,  Biochemical and clinical skin parameters | t-test or nonparametric test. | Polluted environment had a negative effect on skin quality. |
| Liu et al. (2018) | Beijing, China | To investigate if a possible link exists between air pollution and acne vulgaris. | N=59325 | Outpatient records of acne patients,  Daily air pollution parameters for PM_10_, PM_2.5_, SO_2_, and NO_2_ | Generalized additive model (GAM) | Increased concentrations of ambient PM_2.5_, PM_10_, and NO_2_ were significantly associated with increased numbers of outpatient visits for acne vulgaris. |
| Dreno et al.  (2019) | France, Germany, Italy, Brazil, Canada and Russia | To assess the most involved exposure factors in acne. | N=6679 | Demographic characteristics,  Nutrition and nutritional supplements,  Occupational factors,  Medications,  Pollutants,  Psychosocial and modern lifestyle factors,  Weather conditions | Multiple logistic regression analysis. W | Nutrition, pollution, stress and harsh skin care, as well as climate and sun exposure may be considered the most frequent factors related to acne. |
