# Supplementary material for: A Review of Advancement on Influencing Factors of Acne: An Emphasis on Environment Characteristics
Source: Front Public Health. 2020 Sep 17;8:450. doi: 10.3389/fpubh.2020.00450 (PMC7527424; doi:10.3389/fpubh.2020.00450)
Supplement: Supplementary file 3 [file Table_3.DOCX]

**Table 3 Summary of selected studies between acne and social environmental factors**

| **Author and year** | **Location** | **Aim of the study** | **Sample** | **Variables of the study** | **Statistical method** | **Main results** |
| --- | --- | --- | --- | --- | --- | --- |
| Cohen-Cole E et al. (2008) | the United States | To investigate whether “network effects” can be detected for health outcomes that are unlikely to be subject to network phenomena. | N=4300-5400 | Demographic characteristics,  Skin problems, | Logistic regression analysis | A friend's acne problems increased an individual's odds of acne problems. |
| Yousaf A et al. (2020) | West Virginia, the United States | To characterize the influence of social media use on acne treatment. | N=130 | Demographic characteristics,  Patient-reported acne severity,  Use of social media guidance for acne treatment,  Improvement of acne with social media suggestions | Descriptive frequencies | 45% patient consulted social media for acne treatment advice (54% of women vs 31% of men), social media influences acne treatment, but only 31% of participants consulting social media made changes fully aligned with the American Academy of Dermatology (AAD) clinical guidelines. |
| Borba AJ et al. (2019) | Los Angeles, California | To determine the accuracy, quality, viewer engagement, and viewer experience of acne videos on social media. | N= 120 videos | Videos from YouTube | t-tests | Viewers seeking video-based educational content on acne are exposed to significantly inaccurate and low-quality information |
