# Supplementary material for: A Review of Advancement on Influencing Factors of Acne: An Emphasis on Environment Characteristics
Source: Front Public Health. 2020 Sep 17;8:450. doi: 10.3389/fpubh.2020.00450 (PMC7527424; doi:10.3389/fpubh.2020.00450)
Supplement: Supplementary file 4 [file Table_4.docx]

**Table 4 Summary of selected studies between health and built environment**

| **Author and year** | **Location** | **Aim of the study** | **Sample** | **Variables of the study** | **Statistical method** | **Main results** |
| --- | --- | --- | --- | --- | --- | --- |
| Ribeiro et al. (2019) | Porto, Portugal | To investigate the relationship between built environment and obesity. | N=803 | BMI,  The residential address of participants,  Characteristics of the socio-economic and built environment (neighbourhood socio-economic deprivation, dwelling density, proportion of mixed-use buildings,  street connectivity, pedestrian access to non-residential destinations and green space) | Generalized additive  models (GAM) | The study showed an association between socio-economic deprivation score, as well as the presence of fast-food restaurants at a walkable distance from the residence and obesity. |
| Dwicaksono et al. (2018) | New York | To assess whether geographical variations of acne are attributable to the built environment. | N=680 | Food Environment Variables,  Physical Activity, Environment Variables | Ordinary least squares (OLS) models,  Geographical weighted regression (GWR) | The higher the density of fast-food restaurants, the higher the obesity rate among middle school students in the southeastern United States. |
| Xu et al. (2015) | Utah | To assess the associations between built environments and individual odds of overweight and obesity. | N=21961 | Individual socio-demographic characteristics, Behavioral factors,  Health conditions with zip code,  Street connectivity,  Walk score,  Distance to parks,  Distance to food environment | Multilevel logistic models | The odds of being overweight and obese were negatively correlated with the distance to the park and positively correlated with the number of fast-food restaurants. |
| Howard et al. (2016) | England | To assess the associations between sales of unhealthy foods in supermarkets and weight status of children. | N=6517 | Weight status,  Food sales,  Demographic co-variates | Linear regression | There was a positive correlation between the sale of unhealthy foods and prevalence of overweight and obesity among children. |
| Poelman et al. (2018) | the Netherlands | To determine the relation between fast-food outlet density (FFD) and the individual risk for cardiovascular disease. | N=2472004 | Incidence of cardiovascular disease,  Fast-food outlet density (FFD) within network buffers (500m, 1000m, 3000m) around an individual address | Logistic regression analyses | Compared to areas without fast food restaurants, the incidence of cardiovascular disease and coronary heart disease was significantly higher in a 500m buffer zone with one or more fast food restaurants. An increase in FFD within 1000m was associated with a significant increase in the incidence of cardiovascular disease and coronary heart disease. Evidence for 3000m buffer or the incidence of stroke and heart failure was less obvious. |
| Helbich et al. (2018) | the Netherlands | To investigate whether the availability of green space and blue space within people's living environments and living next to the coast are protective against suicide mortality. | N=1190 | Suicide deaths per  municipality for 2005–14,  The proportion of green and blue space per municipality (calculated as a percentage) | Bayesian hierarchical Poisson regressions | Compared with cities with fewer green spaces, cities with larger or moderate green spaces had a lower risk of suicide. |
| Tomita et al. (2017) | South Africa | To explore the potential role of green living environment in alleviating sudden depression in South Africa. | N=11156 | Individual characteristics,  Normalised difference vegetation index (NDVI) | Logistic regression models | The higher the NDVI, the lower the incidence of depression in the middle-income population. |
| Bodor et al. (2010) | New Orleans | To assess associations between access to food retail outlets and obesity in New Orleans. | N=3925 | Individual characteristics,  Obesity prevalence | Hierarchical linear models | Fast food restaurant and convenience store access increased the risk of obesity. |
| Astell-Burt et al. (2013) | Australia | To investigate whether people who lived in neighbourhoods including lots of green space were likely to spend more time outdoors and, subsequently, were more likely to report a case of skin cancer. | N= 267072 | Percentage green space,  Number of hours  spent outdoors in a  week,  Number of moderate-to-vigorous physical activity (MVPA) sessions in a week,  Medically diagnosed skin cancer | Multilevel logit regression | Those with 80% green space were 9% more likely to develop skin cancer than those with 0-20% green space. |
| Liu et al. (2019) | China | To explore biopsychosocial pathways linking residential greenness exposure to depression in the China. | N=21086 | Depression Scale (CES-D),  Resident's exposure to greenspace,  Mediators: stress reduction, physical activity promotion and social, cohesion promotion,  Population density and average income | Multilevel linear models | There was a negative correlation between green space exposure and depression in residential areas. |
| Helbich et al. (2019) | Beijing, China | To examine associations between exposure to green  and blue spaces and geriatric depression in Beijing, China. | N=1190 | Green and blue space per neighborhood,  Geriatric Depression Scale (GDS-15) | Multilevel regressions | In China, green and blue spaces in streetscapes can prevent depression in older people. |
| O'Callaghan-Gordo et al. (2019) | Spain | To study the association of access to green spaces and surrounding greenness with obesity in Spain. | N=2354 | BMI and Waist-Hip Ratio (WHR),  Normalized Difference Vegetation Index (NDVI) | Logistic mixed-effects models | In Spain, greenery may reduce a woman's risk of being overweight/obese. |
| Huang et al. (2019) | China | To investigate the relationship between greenness and obesity in Chinese adults and to assess air pollution and physical activity as mediators of the relationship. | N=24845 | Waist circumference (WC),  BMI,  Normalized Difference Vegetation Index (NDVI) | Two-level logistic and generalized linear mixed regression models | Higher levels of community greening were associated with lower obesity, especially among women, the elderly, and those with lower household incomes. |
| Dons et al. (2018) | European cities (Antwerp, Barcelona, London, Oerebro, Rome, Vienna, Zurich) | To associate transport mode and BMI cross-sectionally | Cross-sectional (N = 7380) and longitudinal (N = 2316) | Individual characteristics,  BMI,  Transport mode | Linear mixed-effects modeling | People lower their BMI when starting or increasing cycling, demonstrating the health benefits of active mobility. |
| Avila-Palencia et al. (2018) | European cities (Antwerp, Barcelona, London, Oerebro, Rome, Vienna, Zurich) | To evaluate the association between different transport modes use and several health and social contact measures. | N=8828 | Individual characteristics,  BMI,  Short Form (SF-36),  Stress Scale (PSS-4),  Transport mode | Mixed-eﬀects logistic regression models,  Linear regression models | Walking and bicycle use was associated with good self-perceived health |
| Mattisson et al. (2015) | Southern Sweden | To investigate the relation between commuting time and mode, and social participation and general trust in other people as measures of social capital. | N=400 | Individual characteristics,  Commuting Mode and Time,  general trust in other people | Poisson regression | In contrast, public commuting was not significantly associated with decreased social capital measures except among long-duration commuters, who reported lower social participation. |
| Martin et al. (2014) | British | To explore the relationship between active travel and psychological wellbeing. | N=17985 | Individual characteristics,  Travel mode choice,  Commuting time,  36-point Likert scale | Fixed effects regression | Active travel was also associated with reductions in the odds of experiencing two specific psychological symptoms when compared to car travel. |
| Frank et al.  (2004) | Atlanta | To evaluate the relationship between the built environment around each participant's place of residence and self-reported travel patterns (walking and time in a car), body mass index (BMI), and obesity | N=10878 | Individual characteristics,  BMI,  Minutes spent in a car,  Kilometers walked,  Age,  Gender,  Income,  Educational attainment | Logistic regression models | Land-use mix had the strongest association with obesity, with each quartile increase being associated with a 12.2% reduction in the likelihood of obesity across gender and ethnicity. Each additional hour spent in a car per day was associated with a 6% increase in the likelihood of obesity. Conversely, each additional kilometer walked per day was associated with a 4.8% reduction in the likelihood of obesity. |
| Mobley et al.  (2005) | the United States | To estimate body mass index (BMI) and the log of 10-year coronary heart disease (CHD) risk as a function of the built environment and socioecologic measures. | N=2692 | Individual characteristics,  BMI,  Built environment,  Socioecologic characteristics | Regression analysis models | The built environment and socioecologic characteristics of financially disadvantaged women were associated with BMI and CHD risk. |
| Xu et al.  (2009) | China | To investigate the association of residential density with overweight among adolescents in an urban area of China. | N=2375 | Individual characteristics,  BMI,  Residential density | Mixed-effects logistic regression models | Residential density was positively associated with overweight among urban Chinese adolescents. |
| Gao et al.  (2016) | Xi’an, China | To assess the associations between built environmental characteristics and health-related quality of life (HRQOL) in an adult population in Xi’ an, China. | N=1608 | Individual characteristics,  Self-reported Neighborhood Attributes (NEWS-A),  Health-related Quality of Life (SF-12) | Population-averaged model | In the new neighborhoods, increased perceived diversity, safety, and esthetics were significantly associated with higher physical and mental well-being. |
| Ribeiro et al  (2019) | Porto Metropolitan Area | To identify areas of elevated prevalence of childhood obesity in the Porto Metropolitan Area and analyse the contribution of the built and socio-economic neighbourhood environment to explain geographic variations. | N=5203 | Individual-level characteristic,  BMI,  Characteristics of the socio-economic and built environment | Generalized additive models | It was possible to identify neighbourhoods with elevated prevalence of childhood obesity and to suggest that targeting such high-priority neighbourhoods and their environmental characteristics may help reduce childhood obesity. |
| Dwicaksono et al.  (2018) | New York | To assess whether these geographical variations are attributable to the built environment. | N= 680 school districts | Food environment variables,  Physical activity environment variables | Ordinary least squares regression models,  Geographical weighted regression models | Higher fast-food restaurant density was significantly associated with higher obesity rates among middle/high school students. |
| Xu et al.  (2014) | Utah | To examine the associations between neighborhood built environments and individual odds of overweight and obesity after controlling for individual risk factors. | N=21961 | BMI,  Socio-demographic attributes,  eighborhood built environment factors | multilevel modeling (MLM) | At the zip code level, poverty rate and distance to parks are significant and negative covariates of the odds of overweight and obesity; and at the county level, food environment is the sole significant factor with stronger fast food presence linked to higher odds of overweight and obesity. |
| Saluja et al.  (2020) | NSW | To investigate the role of fast-food outlet density as a novel environmental risk factor for myocardial infarction in the Hunter region. | N=3070 | Fast-food outlet density (FFD),  Incidence of myocardial infarction | Weighted linear regression | Fast-food outlet density was positively associated with incidence of myocardial infarction in both rural and metropolitan areas of NSW. |
