# Supplementary material for: A Review of Advancement on Influencing Factors of Acne: An Emphasis on Environment Characteristics
Source: Front Public Health. 2020 Sep 17;8:450. doi: 10.3389/fpubh.2020.00450 (PMC7527424; doi:10.3389/fpubh.2020.00450)
Supplement: Supplementary file 5 [file Table_5.DOCX]

**Table 5 Summary of the details about the influence of the environment factors**

| **Environment factors** | | **The operational definitions of the environment determinants** | **Is there any dose-response for environment determinant** | **Study area** |
| --- | --- | --- | --- | --- |
| Natural environment | Temperature and humidity | Average temperature and average humidity | Acne occurrence was significantly more frequent in hot or humid regions. | France; Germany; Italy; Brazil; Canada; Russia |
|  | Sun exposure | Frequency and dose of sun exposure | Acne was significantly more frequent in individuals with moderate or intensive sun exposure due to their work or daily activities. | France; Germany; Italy; Brazil; Canada; Russia |
|  | Air pollution | Frequency and dose of PM_2.5_, PM_10_, and NO_2_ | Increased concentrations of ambient PM_2.5_, PM_10_, and NO_2_ were positively correlated with numbers of outpatient visits of acne vulgaris over the past 2 years. | Beijing, China |
|  | Mineral oils or halogenated hydrocarbons | Frequency and dose of mineral oils or halogenated hydrocarbons | The vast majority of people with acne were significantly more exposed to tar, solvent emanation and crude oil or oil emanation than people without acne | France; Germany; Italy; Brazil; Canada; Russia |
| Social environment | Social network | The interaction between individuals of the society | A friend's acne problems increased an individual's odds of having acne problems | the United States |
|  | Social media | Virtual communities and online platforms that people use to share and exchange ideas | Only 31% of participants consulting social media made changes fully aligned with the American Academy of Dermatology (AAD) clinical guidelines. | West Virginia, America |
| Built environment | Population density | The average number of people living on land per unit area. | Residential density was positively associated with overweight. | Nanjing, China;  Tennessee, America;  Kansas, America |
|  | Food stores | The type and number of food stores in the residential area. | The number and density of fast-food restaurants around the residence were positively correlated with obesity. | New York;  Utah;  the Netherlands;  New Orleans;  New South Wales, Australia;  Kansas, America; |
|  | Green spaces | The proportion of green spaces in the residential area | Green space was positively correlated with mental health. | the Netherlands;  South Africa;  Beijing, China;  Guangzhou, China  Plovdiv, Bulgaria; |
|  |  |  | Green space was negatively correlated with overweight/obesity. | China;  Spain; |
|  | Other built environment characteristics for transport | The choice of the transport mode | Active travel (walking or cycling) was negatively correlated with overweight/obesity. | Antwerp; Barcelona; London; Örebro; Rome; Vienna; Zurich;  Brisbane, Australia;  Taiwanese, China  Atlanta, Georgia region, America; |
|  |  |  | Active travel (walking or cycling) was positively correlated with mental health. | Antwerp; Barcelona; London; Örebro; Rome; Vienna; Zurich; British; |
|  |  |  | Commuting by car was significantly associated with a higher prevalence of low social participation and low general trust compared with active commuting, and the association increased with the duration of commuting time. | Scania, Sweden; |
